# Designing digital mental health interventions for older adults: a scoping review

**DOI:** 10.1038/s41746-026-02523-7

**Published:** 2026-03-13

**Authors:** Dakshayani Rajappan, Ruoyu Yin, Laura Martinengo, Lorainne Tudor Car

**Affiliations:** 1 School of Social Sciences, Nanyang Technological University Singapore, Singapore, Singapore; 2 Lee Kong Chian School of Medicine, Nanyang Technological University Singapore, Singapore, Singapore; 3 Centre for Behavioural and Implementation Sciences Interventions, Yong Loo Lin School of Medicine, National University of Singapore, Singapore, Singapore; 4 Department of Primary Care and Public Health, School of Public Health, Imperial College London, London, UK; 5 King’s Population Health Institute, Kings College London, London, UK

**Keywords:** Geriatrics, Psychiatric disorders

## Abstract

This scoping review aimed to explore the technical and health content-related features that digital mental health interventions (DMHIs) for older adults should entail to facilitate their future design, development, and implementation. We included peer-reviewed expert opinion papers, experimental studies and their protocols on DMHIs for older adults. We searched PubMed, Embase, PsycINFO, Web of Science and Google Scholar. A total of 98 studies were included, comprising 81 experimental studies and 17 expert opinion papers. The DMHIs reported in experimental studies and their protocols included mobile apps, online platforms, and videoconferencing tools, targeting depression, anxiety and grief. However, experts highlighted three main challenges faced by older adults: functional limitations, limited digital literacy, and restricted access to technology. This review provides considerations for the development of future DMHIs, including co-design with older adults, content adaptation, gamification, stakeholder involvement, and privacy and data security. Further research is needed to evaluate these considerations for real-world settings.

## Introduction

About 14% of older adults aged 60 years and above worldwide have a mental disorder^1^. Common mental illnesses in older adults include depression, anxiety, schizophrenia, bipolar disorder and substance use disorder^2^. Important risk factors associated with late-life mental disorders are social isolation, adverse life events, cognitive impairments, and physical disabilities^3^. Mental health conditions in older adults remain largely untreated, with a global treatment gap of 50%, largely because of stigma and stereotypes, lack of human resources, and financial considerations^4,5^. Digital mental health interventions (DMHIs) are becoming increasingly popular due to their ease of access^6^. DMHIs refer to technology-based interventions that aim to prevent, educate, or treat mental health conditions, which are delivered fully digitally or through blended formats where interventions can be self-guided and integrated with healthcare professional support^7^. Research shows that DMHIs are effective in improving mental health and promoting users’ well-being in youth and the general population^8,9^. Common applications of DMHIs include communication with mental health professionals via videoconferencing or messaging services, online peer communities, digital mental health education, and remote access to counselling services^10^. DMHIs may also incorporate self-monitoring and automated tailoring features, allowing users to track progress and receive customised support concurrently^11^. DMHIs via mobile apps or websites can deliver cognitive behaviour therapy (CBT) and interpersonal therapy^12^.

Older adults are increasingly adopting digital technologies^13^, indicating the potential for digital health interventions in this population. However, older adults’ adherence to online interventions appears to be lower than younger populations^14^. Older adults face barriers to using digital health interventions, such as a lack of digital literacy, technology anxiety, and concerns around the inability to express their problems eloquently through online platforms compared to face-to-face consultations^15^. As a result, many of them still prefer in-person treatments^16^. Additionally, older adults may benefit from content relevant to their needs and interests, such as maintaining independence, coping with loss, social isolation, and managing functional decline, but these topics are often lacking in current DMHIs^17^. Older adults also struggle with complex attentional tasks, requiring concise and minimal content woven into educational components in the interventions^9^. To address these barriers, DMHIs need to be tailored to older adults’ needs and preferences.

While DMHIs have been shown to be effective in the general population^18^, these interventions mostly do not consider older adults’ needs and preferences, nor are they specific to older adults^19^. Meanwhile, a systematic review on older adults’ views and experiences with DMHIs demonstrated that older adults’ adoption, engagement and use of DMHIs were influenced by their design^20^. To inform future research in this area, this review aimed to synthesise design features of the DMHIs for older adults evaluated in experimental studies and expert opinion papers that provide a broader vision on design, technology innovations, and future-oriented recommendations. Consistent with Joanna Briggs Institute guidance for scoping reviews^21^, we use these diverse evidence sources to map a broad and evolving field, in which conceptual and empirical work coexist. We were interested in different aspects of the DMHI design, including content, technical, structural, and engagement features. To this end, we performed a scoping review of expert opinion articles, experimental studies and their protocols evaluating the effectiveness, feasibility, and implementation of DMHIs for older adults.

## Results

**Fig. 1:**
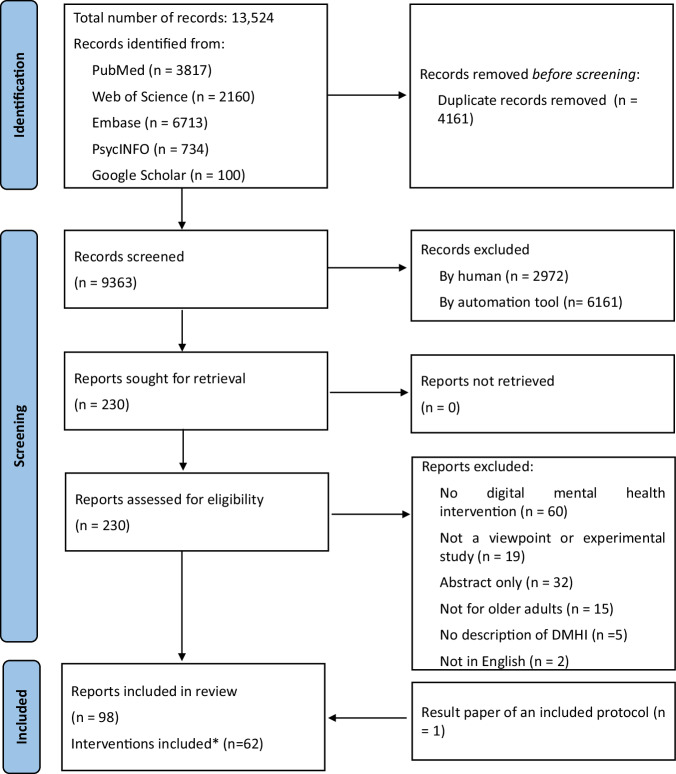
**PRISMA flow diagram of the screening process**. *In experimental studies.

A total of 13,524 papers were retrieved from the databases. Upon removal of duplicate records, 9363 papers were screened. 2972 papers were excluded by the reviewers, while 6161 were excluded by ASReview. A total of 230 full-text records were assessed, and 98 papers met the eligibility criteria and were included (Fig. 1)^22–119^. Eighty one of the included papers were experimental studies or their protocols, reporting 62 interventions, while 17 were expert opinion papers.

Table 1 presents a summary of the studies included in this review. Most of the included interventions were from Asia (*n* = 21, 33.9%), Europe (*n* = 19, 30.6%), and North America (*n* = 11, 17.7%). Most studies focused on depression either stand-alone (*n* = 27, 27.6%) or together with another mental health disorder (*n* = 44, 44.9%).

**Table 1:** Characteristics of included studies

Characteristics	Studies, n (%)
**Total**	**98 (100.0%)**
**Year of Publication**	
2025	11 (11.2%)
2020-2024	73 (74.5%)
2015-2019	13 (13.3%)
2010-2014	1 (1.0%)
**Study Design**	
Experimental studies or protocols	81 (82.7%)
Expert opinions	17 (17.3%)
**Mental Health Target**	
Depression and anxiety	38 (38.8%)
Depression	27 (27.6%)
General mental health	21 (21.4%)
Depression and insomnia	5 (5.1%)
Grief, insomnia, bipolar disorders or other mental disorders	3 (3.1%)
Grief disorder	3 (3.1%)
Depression and grief disorder	1 (1.0%)
**Country of interventions**^**a**^	**n** = **62**
Asia	21 (33.9%)
Europe	19 (30.6%)
North America	11 (17.7%)
Australia	8 (12.9%)
South America	1 (1.6%)
International	1 (1.6%)
Africa	1 (1.6%)

^a^in experimental studies.

Table 2 presents the themes and subthemes in the experimental studies. The included experimental studies provided in-depth descriptions of the DMHI evaluated in the clinical trial. These descriptions focused on the following: (1) characteristics of DMHIs for older adults, (2) mental health therapeutic content in DMHIs for older adults, (3) adaptation of DMHIs for older adults, and (4) findings.

**Table 2:** Themes and subthemes from included experimental studies

Themes	Subthemes	Codes
Characteristics of DMHIs	Delivery channels	Mobile phone
		Computer
		Tablet
		Wearable/ sensor-based tool
		Television
		Gaming device
		Virtual Reality
	Duration and frequency	Duration of a single module
		Frequency of DMHI
		Overall duration of the programme
	Interactivity	Automatic feedback
		Interaction with other users or facilitators
		Interaction with chatbot
	Stakeholder support	Technical assistance
		Therapeutic support
Mental health therapeutic content	Target conditions	Grief
		Depression
		Anxiety
		Sleep disorders
		Other mental disorders
	Therapeutic approach	Cognitive behavioural therapy
		Acceptance and Commitment Therapy
		Psychoeducation
		Positive psychology
		Behavioural activation
		Telepsychiatry
		Problem-solving techniques
		Mindfulness
		Art therapy
		Dialectical behaviour therapy
	Content format	Text
		Audio
		Figure
		Video
		Animation
		Gamification
Adaptations	Adapting duration and deliver timing	Tailored programme duration
		Adjusted session duration
		Brief formats
		Optimised delivery time
	Accessibility	Enhancing features to address visual, motor, and auditory impairments
		Ease of use
		Simple language
		Short content
		Ensuring equitable access for minority groups
	Content	Age-relevant topics
		Older adult–specific narratives
		Older adult–tailored assessments
	Personalisation	Tailoring content individually
		Customising frequency of reminders
Findings	Clinical and implementation outcomes	Effectiveness
		Feasibility and implementation
	User experience	Acceptability
		Facilitators
		Barriers
	Recommendations	Recommendations for future DMHIs

## Experimental studies

### Theme 1: Characteristics of DMHIs for older adults

Supplementary Table 1 presents detailed information on the DMHI and the programme. The DMHIs described in the experimental studies included apps^32,40,53,55,63,64,69,88^, computer software^95,104,112^, online platforms^27,29,35,43–46,51,52,54,76,82,86^, video conferencing^28,41,57,70–72,75,81^, sensor-based symptom monitoring^64,74^, tele-drama^80^, digital games^73^, and virtual environment^89^. The DMHIs deliver psychosocial interventions in an automated, interactive way or serve as virtual collaboration tools for healthcare professionals. These interventions were relayed through various delivery channels, including mobile phones, computers, tablet devices with internet connection, wearables, television, gaming devices, and virtual reality (VR).

The duration of a single module, as well as the overall duration and frequency of the programme, were important components of the intervention programme. The modules duration ranged from five minutes^64^ to three or four hours^57^, with most studies setting a duration of between 20 and 60 minutes. DMHIs were structured, mostly presenting their content in the form of modules that had to be completed in sequence^60,94,105,108,116^ while few allowed users to access the content without a fixed sequence^51,91,93^. The overall duration depended on the number of modules the DMHI intended to cover. Most of them lasted between one and twelve weeks. However, six interventions spanned four to twelve months^38,83,85,101,109,114^, while one DMHI programme lasted only three days^64^.

Interactivity was a common feature in the DMHIs, aiming to promote engagement. Interactivity was mainly achieved through automatic feedback from chatbots, interaction with other users or facilitators, and interaction with chatbots. Automatic feedback from chatbots involved system-generated responses that were triggered by user input without requiring a conversational exchange, while interactions with chatbots constituted dynamic engagement, where users actively communicated in a dialogue format as part of real-time interactions. For example, some DMHIs automatically generated lifestyle suggestions based on diary entries. One study described a sleep intervention that provided recommendations for sleep restriction treatment based on previous weeks’ sleep diaries^94^. Thirteen DMHIs allowed users to interact with each other. One DMHI included a “mailbox” feature where participants could anonymously post questions or share stories, which were visible to others^108^. In another DMHI, there was a WhatsApp group for the participants to discuss with one another and with the facilitator^112^. Four studies utilised chatbots to increase the interactivity of the DMHI. Another protocol collected a holistic profile of users through standard onboarding questions and created a natural dialogue-based chatbot to tackle risk factors of grief and loss accordingly^115^.

**Stakeholder support** to older adults using DMHIs was a key consideration in the intervention delivery, mainly including technical assistance and therapeutic support. Technical assistance was often provided by experienced researchers on the interface of the intervention, and through tending to queries sent by participants via email or a social media platform, which allowed users to receive individualised support^75,101,107^. Additional trainings on digital literacy were sometimes conducted to facilitate older adults’ use of DMHIs^23,50,57,68,85^. In one experimental study, when participants faced technical challenges, social workers would visit their homes to resolve the issues^57^. The research staff in another study called participants every week to help them overcome technical challenges^50^. Therapeutic support entailed input from trained mental health professionals, including providing guidance and feedback on activities completed by participants^46,76,105^. Such support was delivered by various stakeholders, such as therapists^23,29,33,46,76,80,83,97^, clinicians^44,55,60,64,79,84^, coaches^35,93^, nurses^23^, and psychiatrists^95^. For example, in one study, therapists supported participants by providing direction to therapeutic activities, addressing their questions, and offering guidance when they encountered difficulties applying the skills^29^. Therapeutic support also included routine facilitation of communication, with stakeholders guiding conversations on platforms such as ‘Zoom’, ‘WhatsApp’ or ‘LINE’^63,103,104,109,110^. In addition, stakeholders encouraged participants to engage with the DMHIs^35,50,60,84,85^.

### Theme 2: DMHIs’ therapeutic content features

The main target conditions of the DMHIs described in the included experimental studies were grief, depression, anxiety, sleep disorders, or other mental disorders, such as post-traumatic stress disorder^46^, and substance use disorder^50^. These interventions often addressed other well-being outcomes, including fear, loneliness, stress, sensory impairments, resilience, and low mood, along with relationship-related challenges between partners^28,50,54,75,82,86,94,97,108,109^.

The most frequently used therapeutic approach was CBT, a problem-focused approach aimed at reducing emotional distress and increasing adaptive behaviour^23,29,32,46,54,60,68–70,72,75,83–86,91,93,95,97,101,103,112,114,115^. Other studies used other psychotherapeutic approaches like Acceptance and Commitment Therapy (ACT)^53,58,69,82,85,105,108,117^, dialectical behaviour therapy (DBT)^70^, psychoeducation^29,32,33,35,38,50,51,55,57,58,80,83–85,94,101,107–109^, positive psychology^37,45,52,54,57,86,108,110,114^, behavioural activation^33,44,50,107,110,115^, telepsychiatry^117^, and mindfulness^28,37,53,69,71,72,74,78,81,88^. Problem-solving techniques were prioritised for depression interventions, often associated with the use of thought records and breathing exercises^118^. Four studies describing three interventions used art therapy, including two on music therapy^27,43,64^ and another utilising comic creation^25^.

The **content format** varied across DMHIs. The interventions mainly provided text-based information to educate users and guide them through psychotherapeutic exercises^29,35,45,46,52–54,58,76,82,84–86,91,94,105,108,109,114,115,117^. Most interventions also delivered information through audio, images, videos, or animations^27,29,33,35,37,39,45,52–54,58,69–71,74,80–82,84–86,88,91,94,107,117,118^, in the form of animated stories^54,86,94^, voice-overs in a didactic text^84^, and video-guided demonstrations^103,104,118^. In addition, five studies aimed to improve older adults’ mental health through games^25,32,69,73,80^.

### Theme 3: Adaptations of DMHIs for older adults

Of the 62 DMHIs, only 30 explicitly reported adapting the intervention design for older adults, i.e. modifications to the DMHI to enhance the older adult user experience and improve engagement. Four types of modifications were employed in the included DMHIs: duration and delivery timing of the interventions, their accessibility, content, and personalisation. The remaining 32 DMHIs did not report such adaptations. Two DMHIs incorporated feedback from older adults during development^70,118^ and another DMHI employed public patient involvement (PPI)^44^. One additional DMHI was co-designed with older adults^51^.

Few DMHIs adapted **duration and delivery timing** to suit older adults^29,63,70,89,118^. One DMHI extended the length of a single meeting from 60 to 75 min and shortened the duration of the programme by one week^70^. Another DMHI presented the content in short videos of less than two minutes to reduce the cognitive load of older adults^118^. Specifically, one DMHI started its course at noon based on survey findings that older adults were preoccupied in the mornings^63^.

Accessibility of DMHI was improved through adjustments in visual, motor, and auditory features, instructions written in simple language, and provided in short paragraphs, which aimed to improve the usability of the DMHIs by older adults. Examples of vision-enhancing features in included articles were the alteration of display and text size, use of high contrast colour schemes, voice-to-text, and audio descriptors^23,39,70,76^. Examples of motion adjustments included assistive touch and simple controls to be used by older adults experiencing tremors or arthritis^109^. Hearing-enhancing features included the installation of LED flashes for app notifications and subtitles for videos^91^. One intervention included a simple and intuitive design, characterised by sufficiently straight-forward controls to facilitate access to users with minimal familiarity with digital technology^109^. Importantly, the sessions were written for easy comprehension, as demonstrated by an intervention explicitly mentioning language use at an eighth-grade reading level^103^. Another intervention included bite-sized paragraphs in a natural conversational style that was manageable for the older adult, visually appealing, and relatable^115^. To promote equitable access by older adults from ethnic minorities, one DMHI was provided in Spanish in addition to English, although the main menu was still in English^85^. Another DMHI included images of diverse older adult populations^70^.

Nine DMHIs enhanced the **r**elevance of their content to the older adult population^29,51,54,57,60,70,76,89,91^. For instance, case stories, examples, or fictional companions aged 65 and older were incorporated to improve relatability^29,54,60^. Other DMHIs also cover common topics in old age, including retirement, grief and loss^76^, brain health, physical changes, transitions in life^70^, or tailor the assessments to cover domains relevant to older adults, such as cognition, and sleep^51^.

Older adults have diverse characteristics and needs, which necessitate personalisation of DMHIs. The most prevalent personalisation features were content tailoring and reminder customisation. One DMHI used machine learning algorithms to optimise the content and duration of music therapy^64^. An intervention systematically assessed users’ characteristics, needs, and risks, such as physical activity, dietary intake, and cognitive and emotional functioning, before customising the content in every module^101^. Some studies tailored the intervention content according to the users’ conditions and preferences^43,85,97^. Other studies achieved personalisation through joint decision making between users and facilitators, coordinators, or healthcare professionals^76,83^. In another study, with clinician involvement, disease-specific information catered to individual patients was uploaded into their customised patient library^109^. Personalised encouraging messages were also introduced through compliance-promoting features^94,107,114^. Furthermore, the users could schedule a short message service (SMS) to receive useful information and email reminders^114^.

### Theme 4: findings

Thirty-five experimental studies reported clinical and implementation outcomes, user experience, identified challenges and offered recommendations to inform future DMHI development and implementation. Although most studies indicated DMHIs feasible to be implemented in older adults, there were inconsistent findings on their effectiveness. Most studies reported that DMHIs were effective in reducing mental disorder symptoms in the follow-ups^24,27,29,41,43,46,51,53,57,64,66,68–70,73,75–77,81,84,88^. Eight studies did not demonstrate any statistically significant effect compared to baseline or control groups^35,39,45,53,54,58,63,71^, spanning diverse DMHI formats, including a self-guided intervention^54^, ecological momentary assessments (EMA)^39^ and art therapy^23^. Design-related limitations reported in these studies included insufficient adaptation of content for older adults^45^, confusing or difficult-to-understand exercises^53^, and disruptions to daily routines caused by frequent EMA prompts^39^. Three studies reported high acceptability of DMHIs in older adults^43,69,84^. Older adults valued DMHIs that integrated social support or increased their social connections^57,63,73,75,85^, supported their autonomy^50,66^, and were easy to use^32,51^, and protected anonymity^46^. However, they also highlighted barriers, such as difficulties using the digital tool^29,50,57,68,85^, overloaded content and timeframe^29,50,53^, and a lack of personalisation features^39,51^. Lastly, users suggested several improvements for future DMHIs, including personalising the content^39,43,51,53,69,76,82^, simplifying DMHI design^58^, facilitating social connection^43,73^, and incorporating guidance^57,85^.

### Expert opinion articles

Expert opinion articles^22,30,31,42,56,59,90,92,96,98–100,102,106,111,113,119^ included literature reviews (*n* = 8), book chapters (*n* = 2), viewpoints (*n* = 2), Delphi studies (*n* = 1), editorials (*n* = 1), invited perspectives (*n* = 2), and letters to the editor (*n* = 1). The thematic analysis of expert opinion articles resulted in three main themes: (1) types of DMHIs, (2) current challenges, and (3) recommendations (Table 3).

**Table 3:** DMHI features from expert opinion articles classified into themes and subthemes

Themes	Subthemes	Codes
Characteristics of DMHIs	Delivery channels	Computers
		Mobile phones
		Tablets
		Virtual reality
		Wearables and sensors
		Robots
	Digital technology	Artificial intelligence and machine learning
	Types of intervention	Internet-based cognitive behavioural therapy
		Psychoeducation
		Telemedicine
		Ecological momentary assessments
		Mindfulness
Current Challenges	User-related challenges	Functional limitations
		Lack of digital literacy
		Lack of interest
	Technology-related challenges	Digital tools not designed for older adults
		Privacy and data security concerns
		Liability and accountability issues
	Social environmental challenges	Lack of access to mental health services
		Lack of access to technology
		Cultural barriers
		Mental health stigma
Recommendations	Co-design	Engaging older adults in design of the DMHI
	Adaptations	Content
		Accessibility
		Personalisation
		Gamification
		Involvement of stakeholders
	Privacy and security	Privacy and data security

### Theme 1: Characteristics of DMHIs for older adults

Experts discussed different DMHIs for older adults delivered through a variety of different types of digital technologies.

Experts discussed several delivery channels. These included computers^99,100,106,111^, mobile phones^22,30,42,59,90,96,99,100,106,111^, tablets^98,99^, VR^42,99^, wearables and sensors^31,42,59,90,98–100,106^, and robots^42,98,106^. Computer software, websites, and mobile apps were the most commonly mentioned ways of delivering DMHIs. VR was suggested as a means to assess mental health symptoms and cognitive abilities. Such interventions allow practitioners to measure cognitive outcomes such as attention, memory, and executive function, by immersing users in stimulated environments^99^. Remote sensing and wearables can collect users’ data to tailor the treatment based on varying user lifestyles. For example, lifestyle habits may be captured by smartphone cameras, global positioning systems, accelerometers, and thermal sensors^98^. According to experts, wearables such as Apple smartwatches and Fitbit with health and location-tracking could be beneficial for personalised treatment planning^99^. Although less commonly mentioned, robots were proposed as an alternative to human support to address functional challenges among older adults. An editorial suggested using socially assistive robots in areas such as rehabilitation, learning, activities of daily living, and expressing emotions^98^.

The role of emerging digital technologies, including artificial intelligence (AI) and machine learning, in geriatric mental health care was discussed. AI tools can be integrated into mobile apps, robots, or wearables and have the potential for enhancing the effectiveness of mental health care in various ways^22,31,56^. For example, existing AI-based chatbots that employ natural language processing can provide personalised recommendations to users^56^. AI can also facilitate symptom detection, optimise DMHI implementation, monitor effectiveness outcomes, and improve scalability^22^. For example, one perspective paper described DMHIs using machine learning algorithms to predict older adults’ moods and screen for potential depression, providing an opportunity for early detection for those without access to mental health services^100^. Another review mentioned a robotic companion powered by AI designed to reduce loneliness and promote mental well-being in older adults^56^.

The types of interventions discussed by experts included Internet-based CBT, psychoeducation, telemedicine (SMS prompts, telephone therapy, and video therapy)^30,31,90,119^, EMA, and mindfulness^30^. Internet-based CBT^99^ and psychoeducation aim to promote users’ mental health literacy and support older adults’ mental well-being. For example, one Delphi study gathered experts’ opinions on a grief intervention consisting of psychoeducation, which included information about emotional reactions and management of bereavement^92^. Other techniques included in the grief intervention included self-care and identifying changes in daily routines^92^. Telemedicine was seen as allowing older adults to communicate with mental health professionals or peer specialists^98^ remotely, either synchronously or asynchronously. This has the potential to improve access to mental health treatment for older adults who tend not to seek mental healthcare services^31,99^. Videoconference-mediated assessments and EMA^99^ were also seen as having the potential to aid mental health professionals in analysing complex behaviours and monitoring treatment adherence.

### Theme 2: Current Challenges older adults face when using DMHIs

Experts emphasised several challenges older adults may encounter when using DMHIs.

User-related challenges encompassed the limitations that may prevent older adults from optimal use of existing DMHIs, including functional limitations, lack of digital literacy, and lack of motivation. Functional limitations such as impaired hearing and vision and cognitive decline were highlighted as the main barriers to effective engagement with digital technologies^90,106,119^. Experts highlighted that many older adults born in the baby boomer generation do not use technology^111^. Moreover, older adults were seen as often lacking the experience and skills with digital tools^22^ as well as the motivation to adopt new technologies^90,106^. As a result of the lack of digital literacy, older adults were also seen as potentially needing more time and effort to learn how to use DMHIs^111^.

In addition, experts noted technology-related challenges affecting the use of DMHIs by older adults, mainly due to the scarcity of digital tools designed for them. One paper mentioned, “digital tools often require too many clicks to generate simple functions and use icons and symbols unfamiliar to older adults”^90^. Despite their potential, AI systems may be less effective for older adults if this population is underrepresented in their training datasets^42,56^. Additionally, DMHIs often fail to address older adults’ cognitive barriers in attention, memory, and motivation while failing to take advantage of older adults’ procedural memory^90^. Privacy concerns were recognised as significant barriers to DMHIs adoption, particularly among older adults, who may be more reluctant to engage with digital health solutions due to fears about data breaches or unauthorised access to their personal information^31,42,56,98,99^. Additional concerns were raised about the potential compromise of privacy and security during remote consultations in a resource-limited setting^113^. Lastly, liability and accountability issues were raised for DMHIs integrating AI. As AI may make mistakes in diagnosis or treatment recommendations, it remains unclear who should be responsible — the AI developer, the healthcare professional, or both^31,42^. These concerns emphasise the need for regulatory frameworks to guide the use of AI in healthcare^42^.

Social and environmental challenges referred to the impact of different social settings on access to mental health services as well as DMHIs. Marginalised older adults, especially those of minority, lower socioeconomic, and lower educational backgrounds, struggle to access traditional in-person mental healthcare due to financial limitations^113,119^. DMHIs were seen as having the potential to provide marginalised older adults access to mental healthcare remotely^119^. However, experts pointed out that older adults often lack access to technology, let alone telehealth services, within their residences^22,31,42,56,111,119^. Furthermore, it was considered that there was a lack of support from the social networks of these users^111^. Additional cultural barriers were identified for ethnic minorities, such as language barriers and hesitancy to disclose personal information, resulting in distrust and disengagement with DMHIs^22,31^. Even if they have access to DMHIs, evidence on the effectiveness of DMHIs in this population remains limited due to a lack of representativeness in research^22^. Mental health stigma could be another barrier for older adults to access mental health services^22,56^, but the anonymity of DMHTs may potentially address this barrier^30^.

### Theme 3: Recommendations for the development of future DMHIs for older adults

The included studies provided recommendations for future DMHIs for older adults, such as co-design, adaptations, and privacy and security.

Experts recommended co-design of future DMHIs, i.e. the active engagement of users in the design process, in this case, older adults. According to expert recommendations, co-designed DMHIs might be better tailored to users’ needs by considering their inherent values, fears, and aspirations^56^^,^^111^. Co-designed DMHIs have shown high levels of engagement among older adults coping with mental disorders. Therefore, experts emphasised the need to incorporate older adults with lived experiences as equal partners in DMHI development research by adopting adult learning theories^98^.

According to experts, five adaptations of DMHIs may improve older adults’ engagement: content tailoring, improved accessibility, personalisation, gamification, and stakeholder involvement.

*Content adaptations* related to tailoring the mental health content in the interventions to better align with older adult experiences^30^. One suggestion was to incorporate comprehensive information on both mental health problems and health risk factors such as obesity, sedentary lifestyle, and unhealthy diet since mental and physical factors heavily influence each other with increasing age^90^. Another expert recommendation was related to including age-specific topics like grief interventions^92^. Experts highlighted the need for the DMHI content to be relevant to the older adult population by allowing users to change default configurations.

*Accessibility* features comprising adaptations in visual, auditory, and tactile interfaces were considered necessary in DMHIs for older adults. Experts recommended that DMHIs include accessibility features such as consistent interfaces and instructions, button shapes and colour schemes, and adjustable image sizes. These modifications would be in addition to existing features of DMHIs, such as short modules, text-to-speech function and adjustable text size. Furthermore, instructions to complete tasks should be easily identifiable, and include demonstration tasks that match the actual tasks’ difficulty^92^. Likewise, experts suggested adopting assistive touchscreen technologies or providing additional devices such as a stylus pen^99^, which might assist older adults in successfully completing online assignments requiring touchscreen devices. Technology devices or functions that can operate without sustained Internet access were suggested for older adults with limited resources^22^. Simplified language and multilingual options were recommended as key strategies to improve accessibility for ethnic minorities and older adults with lower educational levels^56^.

*Personalisation* was another important design feature mentioned by experts and referred to tailoring an intervention to the diverse and individual needs of older adults by selecting individualised topics, tailoring module sequences, and creating personas. Feedback from experts in a Delphi study^115^ recommended that a DMHI should present individualised content, and dynamically adjust the sequence of content throughout the programme^92^. Furthermore, structural adaptation strategies were proposed to be adopted such that the order of modules, length of interventions, and time spent on each topic can be manipulated^92^. Experts established reasonable consensus on these two adaptation strategies while there was less agreement on tailoring coaching styles between users and conversational agents^92^. DMHIs with real-time adjustability, such as just-in-time adaptive interventions, could enhance personalisation^22^. AI further strengthened the personalisation of DMHIs by adapting to individual users’ educational level, cognitive ability, and language^56^. Another suggestion from experts was creating personas, which are fictional characters displaying important characteristics of prospective users^106^. Older adults with lived experiences suggested that aspects such as names, age, gender, educational backgrounds, and life stories could be considered in archetypes^106^. Using personas in the DMHI design process might improve alignment between the intervention and users’ needs.

*Expert*s mentioned that gamification could improve user engagement with the intervention tasks by incorporating gaming elements. An example of such an intervention mentioned by the experts was the ‘Challenger’ app for social anxiety disorder. Treatment goals were integrated into a game, allowing for the customisation of challenges. This led to greater engagement and adherence^100^.

According to experts, implementing DMHIs in older adults required *the involvement of stakeholders*, such as healthcare providers, mental health professionals, or caregivers, to ensure the necessary support when using DMHI and to sustain their motivation when engaging with it in the long run. These stakeholders would provide two modes of support - technical and social. According to experts, technical support in the form of relevant training on how to use DMHIs could improve self-efficacy and digital literacy skills among users^111^. Another form of technical support that was proposed was to conduct instructional training that might allow older adults to better interact with DMHIs^99^. Social support could be achieved through feedback by an assigned therapist throughout the intervention, pre-programmed supportive and educational messages, or peer support through chatrooms and social media^22,106^. For older adults with limited social support, experts highlighted the potential benefits of incorporating social interaction components into DMHIs, such as social networking features and online communities^22,31^. Finally, one literature review recommended that AI to be a complement to human mental health professionals to enhance acceptability by older adults^56^.

*Privacy and security* were seen as important considerations for future DMHIs. On the one hand, DMHIs were considered as a way of improving privacy because of anonymity^99^. Experts emphasised the importance of data protection guarantees, ensuring that users’ personal information remains secure while also allowing them to choose their preferred mode of communication for remote consultations - whether via text, audio or video calls^113^. It was suggested that creating a personal profile should be optional to address older adults’ privacy concerns^22^. Experts believed that DMHIs should align with standards of protection of sensitive patient information^56,99^. For example, PeerTECH, an existing DMHI mentioned by experts, demonstrated strong privacy protections by complying with the Health Insurance Portability and Accountability Act (HIPAA)^99^.

## Discussion

In this scoping review, we compiled and presented design features of the currently evaluated DMHIs as reported in the literature, as well as expert recommendations for future DMHIs for older adults. Currently evaluated DMHIs for older adults target depression, anxiety, grief, and insomnia. They are delivered in short modules with support from healthcare providers and are adapted and personalised to the needs of older adults. Future DMHIs for older adults, in line with expert recommendations, should incorporate technological advances in the delivery of DMHIs, such as VR, wearables and sensors, and assistive robots. In addition, DMHIs should also be co-designed with older adults, consider additional adaptations such as personalisation by creating personas, gamification, and assistive touchscreen technologies for older adult users, and ensure privacy and security safeguards.

Our findings show the importance of involving healthcare providers, caregivers, peers, or the research team to ensure older adults use DMHIs. This is in line with the findings of a recent systematic review that a blended approach combining a DMHI with human support reduced the likelihood of attrition in older adults^120^. These findings can be partially credited to providing the necessary technical support to older adults with varying levels of technological literacy, as evidenced by experimental studies^23,50,57,68,75,85,95,101,105,107^ and expert opinion papers^99,111^. In addition, DMHIs delivered by healthcare professionals, such as online video calls, were perceived as more trustworthy^15^, especially when there was a pre-existing therapeutic relationship^121^. In addition to technical support and professional guidance, we found in an expert opinion paper that encouraging messages from a therapist or on social media may also improve older adults’ use and engagement with DMHIs^106^, which is consistent with existing systematic reviews^122,123^.

Our findings also show the need to make the DMHIs more accessible. Considering the high prevalence of dexterity issues and visual and hearing impairments in older adults, developers of DMHIs for older adults may consider following the Web Content Accessibility Guidelines (WCAG)^124^ to design more accessible DMHIs. The WCAG are globally recognised guidelines published by the World Wide Web Consortium to increase the accessibility and usability of digital content for people with disabilities and older adults^124^. Existing literature shows that digital health interventions lacking accessibility features could be unable to achieve the goal of health management and improving the quality of life in older adults^125^. The lack of accessibility may also lead to the exclusion of older adults who have health impairments and further exacerbate the digital divide between younger and older generations. Pilot testing by older adults and co-design could help identify accessibility issues. However, only one DMHI employed co-design^51^. Two incorporated feedback from older adults during development^70,118^ and another used PPI^44^. Thirty DMHIs reported their adaptations for older adults. This gap calls for greater involvement of older adults in the design of DMHIs and more transparent reporting of the design process. The additional accessibility features highlighted in expert opinions included devices that may help overcome input issues, such as iPad or stylus pens^99,106^, extending the time between the instructions and the start of tasks to consider older adults’ speed of reading and comprehension, and allowing tasks to be skipped to prevent frustration^106^.

Our review included a comprehensive literature search that allowed for identifying literature on a range of DMHIs. We closely followed the JBI, and the PRISMA-ScR guidelines to conduct and report this scoping review. However, the review has some limitations. First, some expert opinion pieces may have been omitted as they are indexed differently from empirical studies. Second, some protocols, expert recommendations, and envisioned uses of DMHIs, including those involving AI, have not yet been empirically validated, which limits the strength of the evidence supporting these recommendations. Nonetheless, the recommendations synthesise the characteristics of evaluated DMHIs, incorporating feedback from participants in the included studies and expert perspectives, which may provide useful insights for developers of future DMHIs for older adults. Third, one reviewer screened the titles and abstracts of the updated literature search in 2025 by using ASReview. To evaluate quality of this approach and minimise the risk of missing eligible studies^126^, a random 5% sample of the studies excluded by the AI tool was screened by the human reviewer. No new eligible studies were found.

**Fig. 2:**
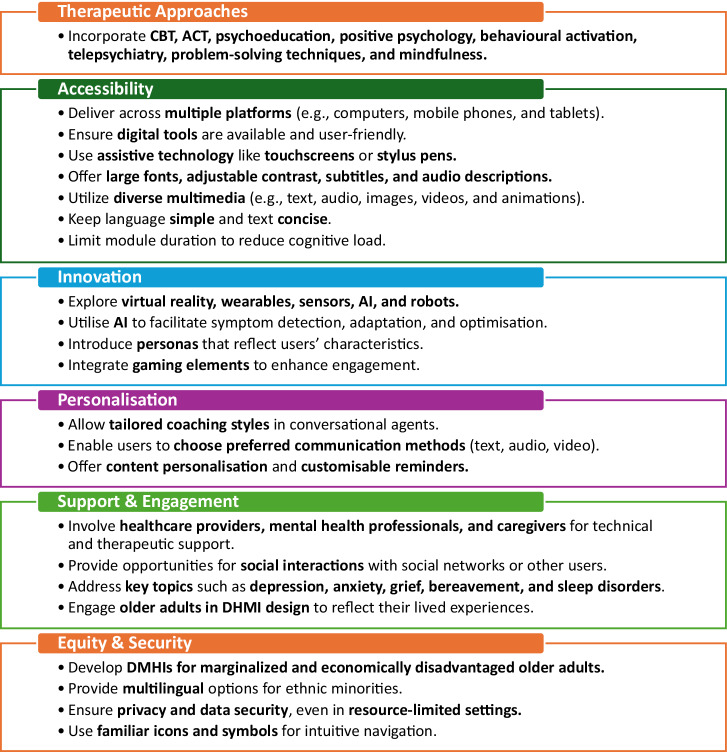
Design considerations for future DMHIs for older adults.

This review provides design considerations for future evaluation, and the design and implementation of DMHIs in older adults (Fig. 2). The identified features represent potential design approaches derived from existing DMHIs and proposed strategies, while awaiting further empirical evaluation. Our findings indicate that DMHIs for older adults should be co-designed, provide content catered to their needs, and include accessibility, personalisation, and gamification features. For example, personalisation can be achieved by adapting content with AI and tailoring chatbot coaching styles to individual user preferences. Support from members of the older adults’ social support network is essential to improve engagement with DMHIs. The implementation of DMHIs requires support by members of the older adult support network, i.e. healthcare providers or family members, to ensure that older adults can navigate the DMHI. Before recommending existing DMHIs to older adults, it is necessary to check whether they cater to their needs in terms of content and design. Additionally, the effectiveness of these DMHIs in improving older adults’ mental health warrants further investigation. It is important that the proposed design features of DMHIs for older adults are robustly evaluated in future studies.

In conclusion, older adults face challenges such as functional limitations, a lack of digital literacy, and insufficient professional support in using digital mental health tools. Experimental studies on DMHIs and expert recommendations for their design included the involvement of healthcare providers in their delivery, adaptations to older adults’ needs and preferences, and personalisation, with AI as a promising option. In addition, experts recommended adopting novel digital technologies for the delivery of DMHIs, co-designing with older adults, and ensuring older adults’ privacy and data security. Our findings identified important design features of DMHIs for older adults that should be considered during development. DMHIs incorporating these proposed design features should be thoroughly evaluated in future studies.

## Methods

The scoping review followed the Joanna Briggs Institute (JBI) scoping review guidelines^21^. We reported this review according to PRISMA-ScR (Preferred Reporting Items for Systematic Reviews and Meta-Analyses Extension for Scoping Reviews) guidelines^127^. The scoping review protocol was registered on Open Science Framework^128^.

### Identifying relevant studies

Published opinion papers and experimental studies (including research protocols) describing DMHIs for older adults that have been peer-reviewed were systematically searched for in PubMed, Embase, PsycINFO and Web of Science on 04/01/2023 and updated on 21/11/2023 and 29/09/2025. We also searched Google Scholar on 18/10/2023 and updated the search on 01/02/2024.

A three-pronged approach was undertaken for the search strategy, consisting of an extensive list of keywords and controlled vocabulary to define “older adults”, “mental health” and “digital health”. The search strategy was developed in consultation with a librarian (Supplementary Note).

### Study selection

This scoping review included expert opinion articles and experimental studies (including study protocols) discussing preferred characteristics of DMHIs for older adults. Expert opinion articles included but were not limited to viewpoints, literature reviews, editorials, and Delphi studies. We also included RCTs, feasibility studies, pilot studies, quasi-RCTs, and their protocols. Protocols without published results were eligible for inclusion because we were interested in the intervention design features, which are usually described in-depth in protocols to improve replicability^129^. To be eligible, papers needed to describe a DMHI aimed at the prevention or improvement of a mental health condition^130^. The interventions included both hybrid or solely online/digital interventions, such as synchronous and asynchronous remote access to therapists via videoconferencing and emails; virtual reality (VR) programmes; computerised, web-based or app-based programmes such as CBT, and online support groups^131^. Reports that mentioned “older adults” were included regardless of the age cut-off, given the variability in definitions. All DMHIs involving older adults were considered eligible. We included articles published in English without geographical limitations. We excluded systematic reviews, qualitative studies, and case reports as these did not primarily focus on outlining specific features, content, or recommendations for digital interventions. Studies focusing on the general or younger populations were also excluded as the scoping review focussed specifically on older adults.

### Data collection

All retrieved studies were uploaded into the EndNote and Zotero reference managers. Duplicate records were identified using duplicate-detection functions in both reference managers and subsequently removed by the reviewers through manual checking. The screening of papers took place in two substages. Firstly, the titles and abstracts of the studies retrieved in the literature searches in 2022 and 2023 were screened by two reviewers (DR and RY) independently and in parallel, assisted by an artificial intelligence (AI) tool called ASReview^132^. We selected naïve Bayes and logistic regression models in ASReview to facilitate the screening. Stopping criteria for ASReview were defined by manually screening about one third of the total number of records and consecutively labelling 1% of the total number of records as “irrelevant”. This approach was in line with prior validation studies of ASReview, which showed that 95% of the eligible records were identified after screening 8% to 33% of the total number of records in four simulation studies^133^. The updated search of the studies published from 2023 to 2025 was screened by one reviewer (RY) on ASReview, applying the same models and stopping criteria as described in the initial screening stage. Next, the two reviewers (RY and DR) thoroughly screened the full text of articles included in the first round. Discrepancies in screening were resolved through discussions between the reviewers or by consulting a third independent reviewer (LM). We have also checked the referenced protocols of the included experimental studies, their associated clinical trial registry entries, and any relevant appendices to ensure that no additional intervention-related information was missed.

### Data extraction and analysis

A Microsoft Excel data extraction form was developed for this study, and included the following information: first author, year of publication, title, type of article, mental health disorder, and type of intervention, involvement of stakeholders, duration and frequency of delivery, therapeutic approach, interactivity, delivery mode, actual content, accessibility features, and data privacy and security. Three reviewers (RY, DR, and LM) conducted data extraction for each included study. All reviewers piloted the extraction form by extracting data from three included studies, and modified it as required according to the reviewers’ feedback. All extracted data was independently verified for accuracy and completeness by a second reviewer, with discrepancies resolved through discussion.

Data was analysed following Braun and Clarke’s thematic analysis methodology^134^. In stage 1, reviewers familiarised themselves with the data extracted by repeatedly reading the content and assessing if there were any underlying themes. In stage 2, initial codes were generated. Specific intervention methods and contents were represented as a short segment of data. In stage 3, overall themes were searched for. The collated codes were assessed and sorted into an overarching theme and were represented in a tabular form. In stage 4, the themes identified were reviewed. The data was thoroughly evaluated to see if there were common patterns, and the validity of the themes in relation to the data extracted was assessed. In stage 5, themes were defined and named. A detailed analysis was conducted on each theme and on any sub-themes that were present, before naming them. Separate themes were developed for experimental studies and expert opinion articles, as the former provided concrete information about implemented or planned DMHIs, while the latter offered broader conceptual reflections and forward-looking recommendations for future development of DMHIs. Maintaining distinct thematic categories preserves conceptual coherence and aligns with scoping review methodology, which encourages mapping diverse evidence streams.

## Supplementary information

Supplementary information

PRISMA-ScR-Fillable-Checklis

## Data Availability

The data supporting the findings of this study are available within the article and its supplementary materials.
