# Supplementary material for: Designing digital mental health interventions for older adults: a scoping review
Source: NPJ Digit Med. 2026 Mar 13;9:264. doi: 10.1038/s41746-026-02523-7 (PMC13036089; doi:10.1038/s41746-026-02523-7)
Supplement: Supplementary file 1 — Supplementary information [file 41746_2026_2523_MOESM1_ESM.pdf]

Supplementary Table 1. Intervention characteristics

Study	Type of intervention	Involvement of stakeholders	Frequency of intervention	Duration of each module	Duration of the programme	Interactivity
**Adler 2024**	app	Yes	3 lessons	30 minutes	Not mentioned	Not mentioned
**Alfaro 2024**	app	Yes	Not mentioned	Not mentioned	Not mentioned	Not mentioned
**Amarti 2022**	Hybrid intervention (face-to-face and Internet-based)	Yes	Weekly	Not mentioned	8 weeks or 16 to 20 weeks	Yes
**Baeza 2024**	app	Yes	3 times per week	60 min	24 weeks	Not mentioned
**Baklouti 2023**	online platform	Yes	Twice a week	80 minutes	8 weeks	Not mentioned
**Berko 2023**	app	Not mentioned	14 lessons over 25 days	20 minutes	25 days	Not mentioned
**Brodbeck 2022**	Website	Yes	10 modules	Not mentioned	10 weeks	Yes
**Calvert 2025**	app	Not mentioned	Once a day	around 20 minutes	4 weeks	Not mentioned
**Catalogna 2025**	app	Not mentioned	Daily	30 minutes	3 week	Yes
**Chan 2024**	app	Yes	One-off	2 hour	Not mentioned	Yes
**Chen 2025**	online platform	Not mentioned	Twice weekly	1 hour	3 month	Not mentioned
**Cho 2022**	app	Not mentioned	Not mentioned	Not mentioned	Not mentioned	Not mentioned
**Chou 2024**	app	Not mentioned	Not mentioned	Not mentioned	4 weeks	Yes
**Chung 2020**	app	Yes	Not mentioned	2 hour training, then use the app at one's own discretion	1 week	Not mentioned
**Cockayne 2011**	Software	Yes	Not mentioned	30-60 minutes	12 weeks	Not mentioned
**Cockayne 2015**	Website	Yes	Weekly	Up to an hour	9 weeks	Yes
**Denkova 2023**	online platform	Yes	Weekly	2 hour	4 weeks	Yes
**Dworschak 2024 a; Dworschak 2024 b**	website	Yes	One module per week	not mentioned	7 weeks	Not mentioned
**Eimontas 2021; Eimontas 2025**	Website	Yes	Not mentioned	Not mentioned	8 weeks	Yes
**Gabarrell-Pascuet 2024**	app	Yes	2 hours/week	Not mentioned	8 weeks	Not mentioned
**González 2024**	app and telephone	Not mentioned	Weekly	90 minutes	6 weeks	Not mentioned
**Gould 2024**	website or DVD	Yes	Weekly	7-37 minutes	4 weeks	Not mentioned
**Heffernan 2019**	Online platform	Yes	Weekly in the first 12 months, then monthly	Not mentioned	Not mentioned	Yes
**Hernandez-Ramos 2024; Borghouts 2022**	app	Yes	At one's own discretion	At one's own discretion	8 weeks in Hernandez-Ramos;	Yes

					12 months in Brghouts	
**Hong2023**	app	Yes	5 days a week	1-2 hour/day	4 weeks	Not mentioned
**Jones 2021**	website	Yes	7 modules over 10 weeks	Not mentioned	10 weeks	Not mentioned
**Juraga 2022**	app and short messages service	Yes	Weekly	2 hours (conventional (face-to-face) group)	7 weeks	Not mentioned
**Keisari 2024**	online platform	Yes	Not mentioned	75 minutes	12 weeks	Not mentioned
**Knaevelsrud 2017**	online platform	Yes	Twice per week	45-minute writing assignments	6 weeks	Yes
**Kwan 2023**	Website, software, app	Yes	Not mentioned	1.5-2 hours	24 weeks	Yes
**Kwan 2023**	Virtual reality	Yes	Two per week	1 hour	6 weeks	Not mentioned
**LaMonica2021**	online platform	Not mentioned	At one's own discretion	at one's own discretion	90 days	Not mentioned
**Lappalainen 2019**	Website	Yes	Not mentioned	Not mentioned	12 weeks	Yes
**Leung 2024**	online platform	Yes	3 online sessions + 1 in-person session	3 or 4 hours	Not mentioned	Yes
**Li 2020**	digital game	Yes	Weekly	3 hours for single player; 1.5 h for multiple player	6 weeks	Not mentioned
**Luo 2024**	online platform	Yes	Eight online sessions and four phone follow-ups	45 min to 60 min per session	8 weeks	Not mentioned
**Meuldijk 2021**	online platform and telephone calls	Yes	STEP 1: 10 module over 13 weeks; STEP2: 2 to 10 sessions in up to 13 weeks	STEP 1 did not mention; STEP 2 one hour	13 - 26 weeks	Not mentioned
**Nakamura 2022**	Mobile app	Yes	4 days a week	Not mentioned	6 weeks	Not mentioned
**Nordgren 2024**	online platform	Yes	Not mentioned		10 weeks	Not mentioned
**Ofosu 2023; De Nys 2024**	website	Yes	Weekly (Ofosu 2023); 3 sessions a week (De Nys)	20 minutes	12 weeks	Not mentioned
**Øverup 2022; Øverup 2022 2024**	Online platform	Yes	Weekly	20-30 minutes	4 weeks	Yes
**Rej 2025**	online platform	Not mentioned	Weekly	90 minutes	8 weeks	Not mentioned
**Roberts 2022**	online platform	Yes	Not mentioned	75 minutes	14 week	Not mentioned
**Sakimoto 2024; Sakimoto 2025**	website	Yes	Once per day, five times per week	10 minutes	a total intervention time of at least 50 min	Not mentioned

**Schuurmans 2016**	Online platform	Yes	Daily	Not mentioned	4 months	Yes
**Scott 2022**	Telephone or web conferencing	Yes	Weekly	Up to an hour	6 weeks	Yes
**Shapira 2021 a; Shapira 2021 b; Shapira 2021 c**	Software, mobile app, and phone call	Yes	Twice-weekly	60-90 minutes	7 weeks	Yes
**Siddiqui 2025**	app	Yes	Not mentioned	the duration of this video was approximately 5 min	3 days	Not mentioned
**Simila 2018**	app	Yes	Not mentioned	1–3 minutes	Not mentioned	Not mentioned
**Song 2024**	app	Yes	Not mentioned		Not mentioned	Yes
**Titov 2016; Staples 2016; Read 2020**	online platform	Yes	One lesson every 7–10 days		8 week	Not mentioned
**Uppsala University 2024**	hybrid (face-to-face and telephone)	Yes	Not mentioned	not mentioned	5 sessions	Not mentioned
**Van Schooten 2021**	Online platform	Yes	Weekly	30 minutes	12 months	Yes
**Van Velsen 2020**	Smartphone app and online platform	Yes	Not mentioned	Not mentioned	Not mentioned	Yes
**Walton 2022**	online platform	Yes	Weekly	60 minutes	10 weeks	Not mentioned
**Weill Medical College of Cornell University**	app	Yes	Not mentioned	not mentioned	Not mentioned	Not mentioned
**Welzel 2021**	Website	Yes	Weekly	Not mentioned	8 weeks	Yes
**Witlox 2018; Witlox 2021**	Website	Yes	Not mentioned	Not mentioned	12 weeks	Not mentioned
**Xiang 2023 a; Xiang 2023 b; Xiang 2023 c; Kayser 2023; Xiang 2024; Xiang 2025 a; Xiang 2025 b; Xiang 2025 c; Xiang 2025 d**	Website	Yes	Weekly	20 minutes per program; 2 minutes per video	10 weeks	Yes
**Yang 2022**	mobile app	Yes	5 days a week	App accessible from 12:00 to 20:30	8 weeks	Yes
**Ying 2021**	app	Yes	5 modules in 5 weeks	Not mentioned	5 weeks	Not mentioned
**Zhang 2025**	digital platform, wearable	Yes	Not mentioned	30 to 40 minutes	8 weeks	Not mentioned

Supplementary Note. PubMed Search Strategy

Older adult terms: elderly[Title/Abstract] OR senior[Title/Abstract] OR seniors[Title/Abstract] OR "older adult"[Title/Abstract] OR "older adults"[Title/Abstract] OR "older person*"[Title/Abstract] OR "older persons"[Title/Abstract] OR geriatric[Title/Abstract] OR geriatrics[Title/Abstract] OR aged[MeSH Terms] OR aged, 80 and over[MeSH Terms]

Mental health terms: "mental health"[Title/Abstract] OR "mental well-being"[Title/Abstract] OR "mental wellbeing"[Title/Abstract] OR "mental disorder*"[Title/Abstract] OR "mental disease*"[Title/Abstract] OR mental health[MeSH Terms]

Digital health terms: app[Title/Abstract] OR apps[Title/Abstract] OR mobile application[Title/Abstract] OR mobile intervention[Title/Abstract] OR mobile based[Title/Abstract] OR cell phone[Title/Abstract] OR smart phone[Title/Abstract] OR smartphone[Title/Abstract] OR mobile health[Title/Abstract] OR mhealth[Title/Abstract] OR ehealth[Title/Abstract] OR digital health[Title/Abstract] OR digital[Title/Abstract] OR internet[Title/Abstract] OR online[Title/Abstract] OR web based[Title/Abstract] OR website[Title/Abstract]
